# Iron acquisition in *Pseudomonas aeruginosa* by the siderophore pyoverdine: an intricate interacting network including periplasmic and membrane proteins

**DOI:** 10.1038/s41598-019-56913-x

**Published:** 2020-01-10

**Authors:** Anne Bonneau, Béatrice Roche, Isabelle J. Schalk

**Affiliations:** 1 CNRS, UMR7242, ESBS, Illkirch, Strasbourg France; 2 Université de Strasbourg, UMR7242, ESBS, Illkirch, Strasbourg France

**Keywords:** Ion transport, Metalloproteins, Iron, Cellular microbiology

## Abstract

Pyoverdine (PVDI) has been reported to act both as a siderophore for scavenging iron (a key nutrient) and a signaling molecule for the expression of virulence factors. This compound is itself part of a core set of virulence factors produced by *Pseudomonas aeruginosa* during infections. Once secreted into the bacterial environment and having scavenged ferric iron, PVDI-Fe^3+^ is taken back into the *P. aeruginosa* periplasm via the outer membrane transporters FpvAI and FpvB. Iron release from PVDI in the bacterial periplasm involves numerous proteins encoded by the *fpvGHJKCDEF* genes and a mechanism of iron reduction. Here, we investigated the global interacting network between these various proteins using systematic bacterial two-hybrid screening. We deciphered a network of five interacting proteins composed of two inner-membrane proteins, FpvG (iron reductase) and FpvH (unknown function), and three periplasmic proteins, FpvJ (unknown function), FpvF (periplasmic PVDI-binding protein), and FpvC (iron periplasmic-binding protein). This interacting network strongly suggests the existence of a large protein machinery composed of these five proteins, all playing a role in iron acquisition by PVDI. Furthermore, we discovered an interaction between the periplasmic siderophore binding protein FpvF and the PvdRT-OpmQ efflux pump, also suggesting a role for FpvF in apo-PVDI recycling and secretion after iron delivery. These results highlight a multi-protein complex that drives iron release from PVDI in the periplasm of *P. aeruginosa*.

## Introduction

*Pseudomonas aeruginosa* is an opportunistic human Gram-negative pathogen considered by the World Health Organization to be an antibiotic-resistant priority pathogen^1,2^. During infections, *P. aeruginosa* faces a stressful environment and must overcome host-defense mechanisms. To survive within the host, *P. aeruginosa* secretes a large number of virulence factors, including the siderophores pyoverdines^2,3^. Siderophores are small organic compounds produced and secreted by bacteria to access iron^4^, a key nutrient essential for bacterial growth and virulence. Strains unable to produce pyoverdines have been reported to exhibit reduced virulence during infections in mice^5^. The role of pyoverdines in the virulence of *P. aeruginosa* has also been ascertained using rabbit and mouse lung infection models^6–8^. Pyoverdines are reported to have a dual role during infection. They are used as a siderophore by *P. aeruginosa* to scavenge iron from the host proteins^5,8^ and also acts as a signaling molecule for the production of two major virulence factors, exotoxin A and the endo-proteinase PrpL^3,9^.

More generally, all fluorescent *Pseudomonas* species produce specific pyoverdines as major siderophores to access iron. These pyoverdines are all composed of a peptide of 6 to 12 amino acids, with a specific sequence, and linked to a chromophore derived from 2,3-diamino-6,7-dihydroxyquinoline^10^. The sequence of the peptide moiety of the different pyoverdines is very different in amino acid composition and in length among pyoverdines and is a determinant specific of each pseudomonads species^10–14^. *P. aeruginosa* strains produce three distinct pyoverdine types (PVDI, PVDII and PVDIII) each characterized by a different peptide chain^15^ and PVDI is the siderophore produced by *P. aeruginosa* PAO1. Molecular mechanisms involved in iron acquisition by pyoverdines have mostly been investigated in *P. aeruginosa* PAO1.

**Figure 1:**
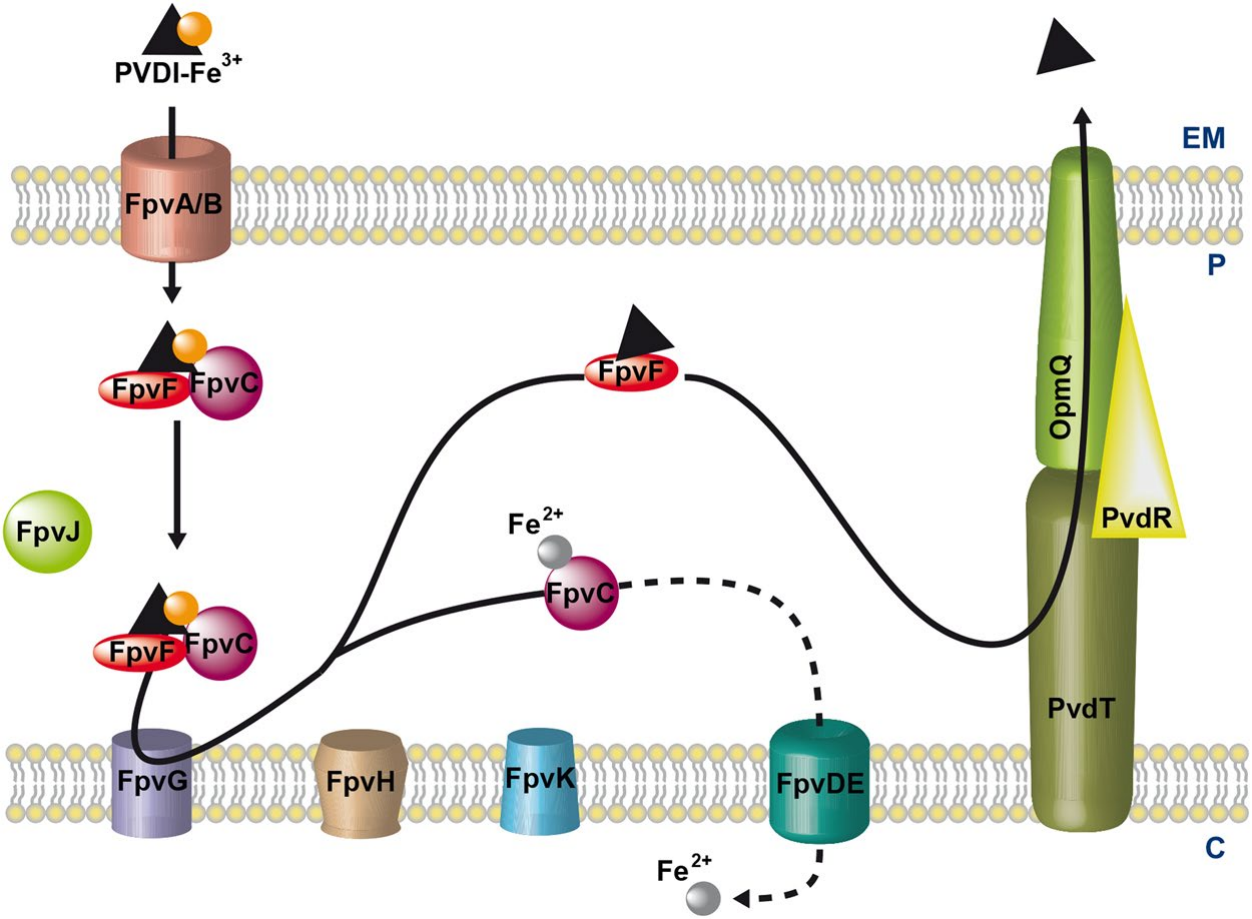
Model of Fe^3+^ uptake by the siderophore PVDI in *P. aeruginosa*. For details, see the Introduction section. EM: extracellular medium; P: periplasm; C: cytoplasm.

PVDI is synthesized by non-ribosomal peptide synthetases in the bacterial cytoplasm^16,17^ and then matures in the periplasm^18^ before secretion into the extracellular medium by the PvdRT-OpmQ ATP-dependent efflux pump^19^. In the bacterial environment, PVDI chelates ferric iron, yielding the PVDI-Fe^3+^ complex^20^. Ferric complexes of this siderophore are then recognized at the bacterial surface and imported across the outer membrane by two specific TonB-dependent transporters, FpvAI and FpvB (Fig. 1), with the TonB-ExbB-ExbD inner-membrane protein complex providing the necessary energy^21–24^. Once in the periplasm, PVD-Fe^3+^ is bound by the two periplasmic proteins, FpvC and FpvF^25^. Iron release from PVDI occurs in the bacterial periplasm and involves no chemical modification of the siderophore but rather iron reduction by the FpvG inner-membrane reductase^26–28^. *fpvG* is localized next to *fpvH*, *fpvJ*, and *fpvK* genes encoding three proteins of unknown function, but of which expression is required for optimal activity of FpvG^28^. Sequence alignment of FpvC revealed that this protein belongs to a group of metal-binding periplasmic proteins^25^, and previous *in vitro* studies of PVDI-Fe dissociation in the presence of DTT showed that FpvC can apparently bind ferrous iron after the reduction step and its dissociation from PVDI^28^. Iron is translocated further across the inner membrane into the cytoplasm by the predicted ABC transporter FpvDE^25^. All four proteins FpvC, FpvD, FpvE and FpvF, which genes are localized next to *fpvGHJK* genes, are also necessary for efficient dissociation of iron from PVDI^28^. After iron release, the apo form of PVDI is recycled into the extracellular medium by the PvdRT-OpmQ efflux pump, with the ability to again chelate Fe^3+^ in the bacterial environment^29,30^. Dimers of the periplasmic protein FpvF are able to bind apo-PVDI^25^ and the recycling of apo-PVDI has been shown to be partially abolished in an ∆*fpvF* mutant^28^, suggesting a role of FpvF in apo-PVDI recycling. Although it has been shown that FpvC and FpvF are able to form a complex that binds PVD-Fe^3+^,^25^, the overall interaction network between all the proteins encoded by the *fpvGHJKCDEF* genes has not been yet investigated.

Here, we characterized the interacting network between *P. aeruginosa* proteins of the PVDI-Fe^3+^ uptake pathway using the bacterial *E. coli* adenylate cyclase two-hybrid system (BACTH) for high throughput interaction screening. The results of BACTH screening revealed (i) an interaction between the two inner-membrane proteins FpvG and FpvH, (ii) an FpvJ-FpvC-FpvF periplasmic complex, and (iii) the ability of the two inner-membrane proteins (FpvG and FpvH) and the three periplasmic proteins (FpvC, FpvF, and FpvJ) to interact in a membrane machinery complex. These complexes were further confirmed by purification and pulldown experiments. We also found that the periplasmic apo-PVDI binding protein FpvF^25^ is able to interact with PvdT, the inner-membrane protein of the PvdRT-OpmQ efflux pump, suggesting a role for FpvF in the transport of apo-PVDI to PvdRT-OpmQ through the bacterial periplasm. Our study has allowed unprecedented deciphering of the interacting network of the various proteins involved in Fe^3+^ release from PVDI in the periplasm of *P. aeruginosa*, linking both membrane and periplasmic proteins.

## Results

### Interaction between FpvG and FpvH and formation of an inner-membrane complex

**Figure 2:**
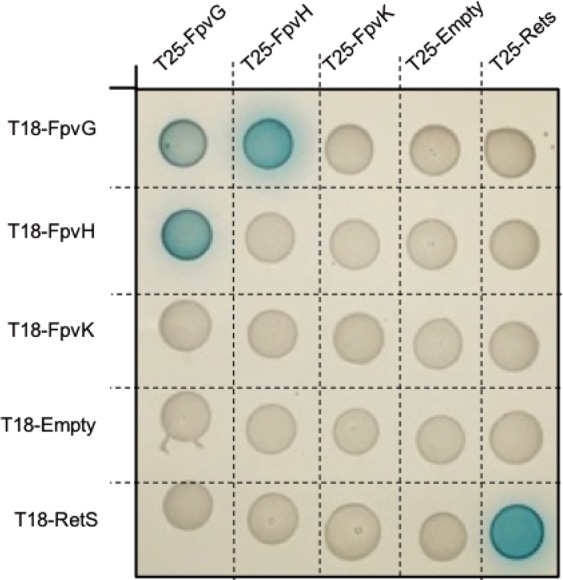
Interacting network with the membrane proteins FpvG, FpvH and FpvK. DHM1 cells producing the protein of interest fused to the T18 or T25 domain of adenylate cyclase were spotted on indicator plates containing X-gal for BATCH screening. Each protein was tested with an empty vector and the RetS protein, which is not related to the PVDI pathway. RetS is able to form dimers and serves as a positive control^37^. The blue color indicates an interaction between the two proteins of interest. The experiment was repeated three times with each time 10 colonies as described in Materials and Methods. A representative image is shown.

Previous studies have demonstrated that expression of the FpvH, FpvK, and FpvJ proteins is required for optimal reductase activity of FpvG^28^. Moreover, genes encoding the FpvG, FpvH, FpvJ, and FpvK proteins are organized in an operon^31^ and it is well recognized that adjacent genes tend to encode interacting proteins^32^. FpvG, FpvH, and FpvK have been predicted to be inner-membrane proteins and FpvJ periplasmic, because of a signal peptide^28^. We deciphered the interacting network between the membrane proteins FpvG, FpvH, and FpvK by performing systematic BACTH screening in *E. coli*, which is based on the reconstitution of adenylate-cyclase activity. The full-length *fpvG*, *fpvH*, and *fpvK* genes were fused to the T25/T18 domains of adenylate cyclase in the two-hybrid vectors. Screening of the possible protein-protein interactions between FpvG, FpvH and FpvK on indicator plates containing X-gal highlighted an interaction between FpvG and FpvH (Fig. 2), whereas no interaction could be observed for FpvK. FpvG interacted with itself, suggesting at least dimerization of this protein (Fig. 2). As FpvG was already been demonstrated to be an inner-membrane protein^28^, we investigated the subcellular localization of FpvH. Cell fractionation experiments showed that FpvH is also an inner-membrane protein (Fig. S1A in Supplemental Material).

**Figure 3:**
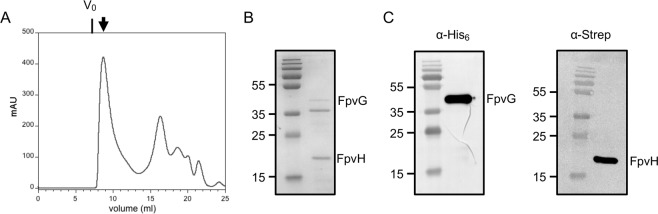
Purification of the inner-membrane FpvG-FpvH complex. (**A**) Size exclusion chromatography analysis of the solubilized and purified FpvG_His6_-FpvH_Strep_ complex on a Superdex 200 10/300 GL column. The dead volume is indicated as V_0_. The arrow shows the fraction containing the FpvG_His6_-FpvH_Strep_ complex. (**B**) SDS-PAGE of the purified complex analyzed by Coomassie blue staining. (**C**) Immunoblot analysis of the purified complex using specific anti-His and anti-Strep antibodies. The molecular mass markers (kDa) are indicated on the left. The predicted size of FpvG _His6_ and FpvH_Strep_ are 46 kDa and 21 kDa respectively (Table S3 in Supplemental Material). Entire SDS-PAGE and blots are shown in Fig. S2 in Supplementary Information.

We expressed FpvG and FpvH proteins with C-terminal His_6_ and Strep-tag sequences, respectively, in *E. coli* to validate their interaction. Bacterial membranes were solubilized in detergent and the complex purified by Strep-trap affinity followed by size-exclusion chromatography (Fig. 3A). The presence of both FpvG and FpvH proteins in the elution peak was confirmed by Coomassie-blue staining and immunoblot analysis using specific anti-His_6_ and anti-Strep antibodies (Fig. 3B,C). Isolation of the FpvG-FpvH complex confirmed the BACTH results and revealed the ability of FpvG reductase and FpvH to interact and form an inner-membrane complex.

### Interaction between FpvJ, FpvC, and FpvF and formation of a periplasmic protein complex

**Figure 4:**
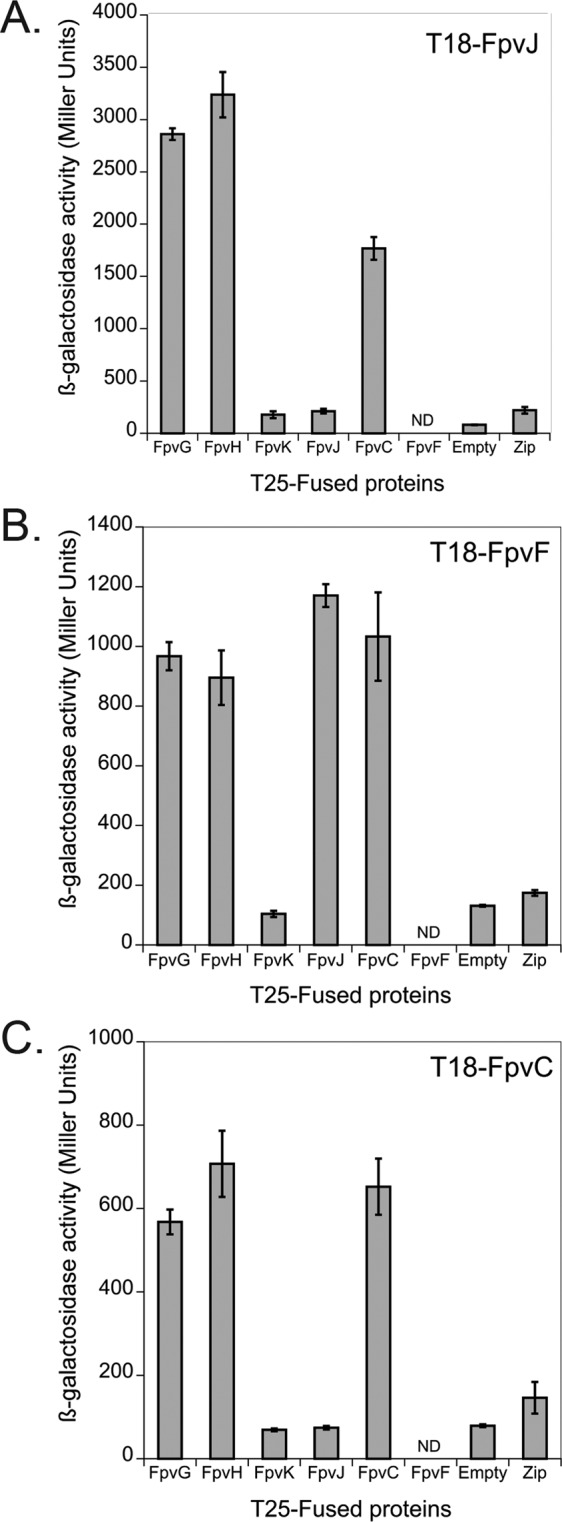
Interacting network with periplasmic (FpvC, FpvF and FpvJ) and membrane proteins (FpvG, FpvH and FpvK). Bacterial two-hybrid assays for proteins were quantified by measuring the β-galactosidase activity, as described in Materials and Methods. Zip, which is not related to the PVDI pathway, served as a positive control^38^. ND: not determined because for FpvF we were unable to obtain the pKT25-FpvF vector. The experiment was repeated three times independently. Error bars represent the standard errors of the means.

FpvC and FpvF are the two periplasmic-binding proteins associated with the ABC transporter FpvDE^25^. Cell fractionation also demonstrated periplasmic localization of FpvJ (Fig. S1B in Supplemental Material). The presence of some FpvJ protein also in the cytoplasmic fraction was probably due to newly synthesized FpvJ, as previously described for periplasmic proteins^33^. Since the indicator plates assay cannot be used to detect interactions between periplasmic proteins, we screened the interactions between FpvJ, FpvC, and FpvF by quantifying ß-galactosidase in liquid cultures (Fig. 4). Concerning the interaction between the three periplasmic proteins, we detected interactions between FpvJ and FpvC (Fig. 4A), FpvF with FpvJ and FpvC (Fig. 4B) as previously shown by Brillet *et al*.^25^, as well as an interaction of FpvC with itself (Fig. 4C). Altogether, our BACTH results show that the three proteins FpvC, FpvF, and FpvJ are able to all interact with each other, suggesting the existence of a periplasmic complex composed of the three proteins.

**Figure 5:**
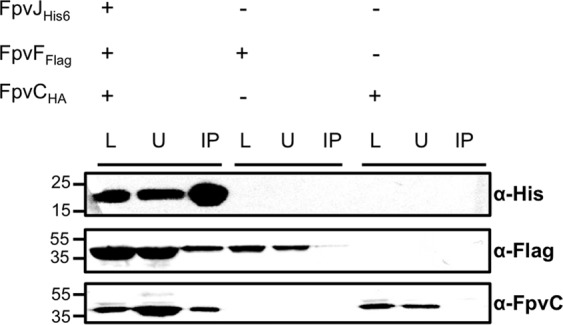
Pulldown assay of the periplasmic FpvJ-FpvF-FpvC complex. Periplasmic fractions of BL21(DE3) cells co-expressing the indicated proteins FpvJ_His6_, FpvC_HA_, or FpvF_Flag_ were incubated with anti-His beads. The loading (L), unbound (U), and immunoprecipitated (IP) fractions were separated by SDS-PAGE and analyzed by immunodetection using anti-His_6_, anti-FpvC and anti-Flag antibodies. The molecular mass markers (kDa) are indicated on the left. The predicted size of FpvJ _His6_, FpvF_Flag_ and FpvC_HA_ are 15 kDa, 33 kDa and 36 kDa respectively. Entire blots are shown in Fig. S3 in Supplementary Information.

We overproduced FpvJ, FpvF, and FpvC in *E. coli*, adding a His_6_, Flag tag, and HA at the C-terminus of each protein, respectively, to confirm the existence of this periplasmic complex. The periplasmic fraction was incubated with anti-His beads. FpvF_Flag_ and FpvC_HA_ co-precipitated with FpvJ_His6_ (Fig. 5). Negative controls showed that neither FpvF_Flag_ nor FpvC_HA_ alone were retained on the anti-His beads. Overall, these results show that FpvJ, FpvC and FpvF can form a periplasmic complex.

### Interaction between the inner-membrane proteins FpvG and FpvH with the periplasmic proteins FpvJ, FpvC, and FpvF

Finally, we also tested the interactions between the inner-membrane and periplasmic proteins. BACTH analysis showed that both FpvG and FpvH membrane proteins interact with the three periplasmic proteins FpvJ, FpvF, and FpvC (Fig. 4). As with the membrane proteins, none of the periplasmic proteins interacted with FpvK in this two-hybrid approach (Fig. 4). Overall, BACTH screening revealed the existence of an interaction network between FpvG, FpvH, FpvJ, FpvF, and FpvC.

**Figure 6:**
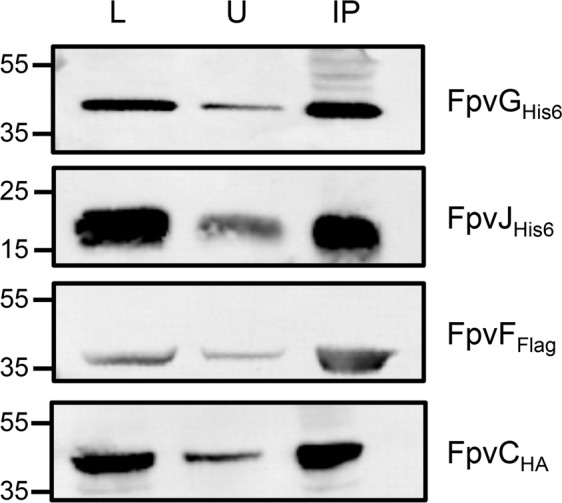
Pulldown assay of the FpvG-FpvJ-FpvC-FpvF complex. Periplasmic fractions of BL21(DE3) cells producing FpvJ_His6_, FpvC_HA_, FpvF_Flag_ and membrane fraction of TOP10 cells producing FpvG_His6_, and FpvH_Strep_ were incubated with anti-Flag beads. The loading (L), unbound (U), and immunoprecipitated (IP) fractions were separated by SDS-PAGE and analyzed by immunodetection using anti-His_6_, anti-FpvC, and anti-Flag antibodies. The molecular mass markers (kDa) are indicated on the left. The predicted size are for FpvG _His6_ 46 kDa, FpvH_Strep_ 21 kDa, FpvJ _His6_ 15 kDa, FpvF_Flag_ 33 kDa and FpvC_HA_ 36 kDa. Entire blots are shown in Fig. S4 Supplementary Information.

We next attempted to isolate all five proteins by pulldown experiments using an anti-Flag resin. The periplasmic fraction of *E. coli* overproducing FpvJ_His6_, FpvC_HA_, and FpvF_Flag_ was incubated with solubilized membranes containing FpvG_His6_ and FpvH_Strep_, and the mixture incubated with an anti-Flag resin. FpvG_His6_, FpvJ_His6_, and FpvC_HA_ co-precipitated with FpvF_Flag_ (Fig. 6). Equivalent results were obtained when fractions were incubated with PVDI-Fe (Fig. S5 in Supplemental Material). None of the non-Flag-tagged proteins were retained on the anti-Flag resin when incubated alone (Fig. S6 in Supplemental Material). We were unable to detect FpvH_Strep_ either due to immunodetection problems or because this protein is not present in the complex. Overall, these results confirm the existence of at least a four-protein complex, linking the inner-membrane FpvG protein and three periplasmic components of the PVDI pathway. In addition, this complex could be isolated in the presence or absence of PVDI-Fe.

### FpvF interacts with the membrane protein PvdT of the PvdRTOpmQ efflux pump

**Figure 7:**
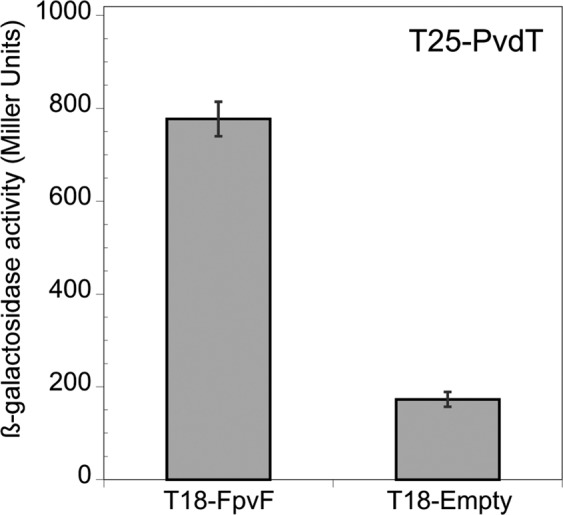
Interaction between the periplasmic FpvF protein and the inner-membrane PvdT protein of the efflux pump PvdRT-OpmQ. Bacterial-two hybrid assays were quantified by measuring the β-galactosidase activity, as described in Materials and Methods. The experiment was repeated three times independently. Error bars represent the standard errors of the means.

Previous studies have shown that FpvF can form dimers that bind apo-PVDI^25^ and that PVDI recycling is altered in a ∆*fpvF* mutant^28^. Based on these observations, it seemed possible that FpvF is involved in PVDI recycling by interacting with proteins of the efflux pump PvdRT-OpmQ. Indeed, our BACTH analysis revealed an interaction between FpvF and the membrane protein PvdT (Fig. 7).

## Discussion

One of the major particularities of the PVDI-dependent iron acquisition pathway in *P. aeruginosa*, and probably conserved among fluorescent Pseudomonads, is that this siderophore delivers iron into the bacterial periplasm, with siderophore-free iron then being transported further by an ABC transporter into the cytoplasm. This mechanism is completely different from that described previously for other siderophore-dependent iron-uptake pathways, such as the enterobactin and ferrichrome pathways in *E. coli*, two archetypes in the field of bacterial iron homeostasis, which deliver iron directly into the bacterial cytoplasm^34^. After the uptake of PVDI-Fe^3+^ across the outer membrane by FpvAI or FpvB^21–23^, iron release from PVDI in the bacterial periplasm requires the FpvGHJKCDEF proteins^28^. Moreover, the molecular mechanism involved implies both iron reduction by FpvG reductase to decrease the affinity of PVDI for the metal and an iron chelator, FpvC^26–28^.

**Figure 8:**
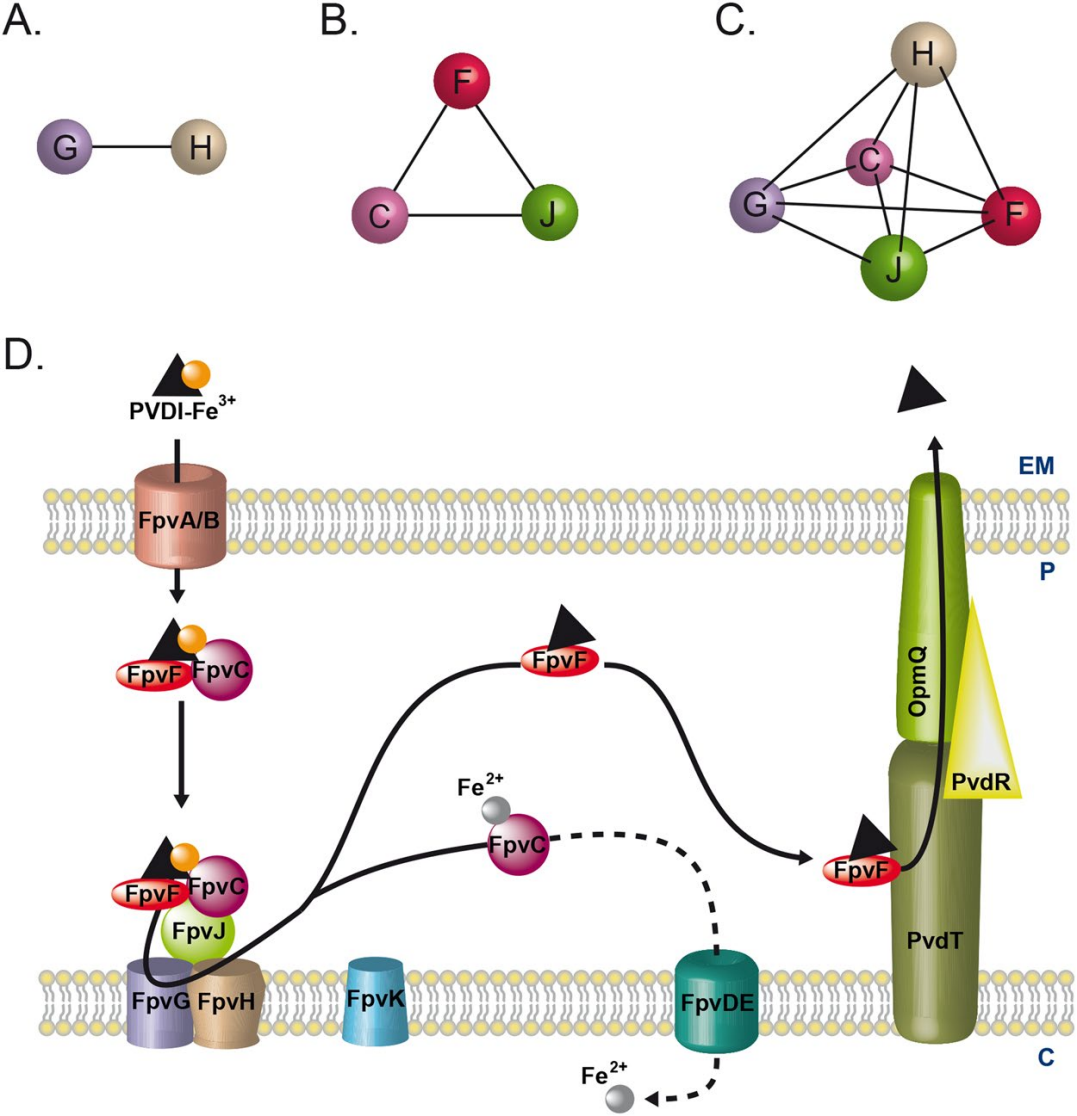
Model of the multi-protein machinery involved in iron acquisition via PVDI in *P. aeruginosa*. (**A**) Interacting network observed between inner membrane FpvG and FpvH proteins. (**B**) Interacting network between periplasmic proteins FpvF, FpvC and FpvJ. (**C**) Interacting network between inner membrane and periplasmic proteins. D. Model of PVDI-Fe^3+^ uptake by *P. aeruginosa* with the protein complexes highlighted. For details, see the Discussion section. EM: extracellular medium; P: periplasm; C: cytoplasm.

We used a systematic BACTH assay to unravel the interactions between these proteins and highlight specific interactions between FpvG reductase, the inner-membrane protein FpvH, and the three periplasmic proteins FpvJ, FpvC, and FpvF (Fig. 8). Although our BACTH screening was carried out using both N- and C-terminal T18/T25 tags, we observed no interactions with FpvK, suggesting that either (i) FpvK does not interact with the three other proteins, (ii) the interactions are transient or of weak affinity, (iii) the interaction requires a third protein partner, or (iv) the interaction is just not detectable by BACTH. Indeed, fusion to the T18 or T25 domains may affect the folding of the protein or prevent interactions^35^.

We biochemically confirmed the interaction between the two inner-membrane proteins FpvG-FpvH by affinity and size-exclusion chromatography, but we still know nothing about the stoichiometry of the FpvG-FpvH complex, except that FpvG is able to form dimers based on the BACTH data. Previously, the *in vivo* kinetics of iron dissociation from PVDI showed that FpvG activity is dependent on FpvH expression^28^. FpvJ and FpvK expression also affect FpvG activity, but clearly to a lower extent^28^. The ability of FpvK to affect FpvG reductase activity, like FpvH and FpvJ, highly suggests that it also interacts with the other inner-membrane proteins.

BACTH screening also showed an interacting network between the three periplasmic proteins FpvC, FpvF, and FpvJ. This complex was validated by pulldown experiments. FpvC and FpvF are two periplasmic-binding proteins associated with the ABC transporter FpvDE. Purified FpvC was shown to chelate ferrous iron in an *in vitro* PVDI-Fe dissociation assay using DTT as the iron reducer^28^. Mass spectrometry approaches under native conditions have shown that FpvF can bind PVDI, and that FpvC and FpvF are both able to form the tetrameric FpvC_2_-FpvF_2_-PVDI-Fe complex in the presence of PVDI-Fe^25^. The existence of FpvC-FpvF-PVD-Fe complexes in *P. aeruginosa* periplasm was confirmed by cross-linking experiments^25^, and we proposed that, after its uptake across the outer membrane, PVDI-Fe forms a FpvC-FpvF-PVDI-Fe complex. Isolation by pulldown experiments of the FpvC-FpvF-FpvJ complex strongly suggests that such a complex may exist in the bacterial periplasm of *P. aeruginosa* and is probably necessary in the mechanisms involved in iron release from PVDI.

The function of FpvJ is currently unknown, but this protein may allow interaction of the FpvC-FpvF-PVDI-Fe complex with the FpvG-FpvH inner-membrane complex to achieve iron reduction and the transfer of ferrous iron from PVDI to FpvC. BACTH screening showed that all three periplasmic proteins FpvJ, FpvC, and FpvF can interact with the two-protein FpvG-FpvH complex in the absence or presence of PVD-Fe, forming an inner-membrane machinery. We were able to isolate four of the five proteins by pulldown assay, confirming the existence of a complex between the inner-membrane reductase, FpvG, and the three periplasmic proteins, FpvF, FpvC, and FpvJ. Immunodetection of FpvH_Strep_ with anti-Strep antibodies revealed non-specific bands of various molecular weights, preventing us from assessing the presence of FpvH in the pulldown assay (data not shown). The exact stoichiometry of this complex is still unknown.

Moreover, FpvDE is the putative ABC transporter that allows the translocation of ferrous iron across the inner membrane into the cytoplasm, and its deletion affects iron acquisition by PVDI^25,28^. We also evaluated the possible interactions of FpvDE with the periplasmic FpvC and FpvF and the membrane proteins FpvG, FpvH, and FpvK, but were unable to detect any interaction (data not shown). However, FpvC probably plays a role in bringing ferrous iron to the permease, FpvE, but further biochemical studies will be necessary to demonstrate this.

Finally, previous studies have demonstrated that FpvF dimers can bind apo-PVDI and apo-PVDI recycling is partially abolished in a ∆*fpvF* mutant^25,28^. We thus investigated whether FpvF can interact with the PvdRT-OpmQ efflux pump involved in PVDI recycling. We found that FpvF interacts with PvdT, the inner-membrane protein of the efflux pump. This result strongly supports the hypothesis that FpvF or FpvF_2_ binds apo-PVDI in a FpvF-PVDI or FpvF_2_-PVDI_2_ complex and brings the apo-siderophore to PvdT for its recycling into the extracellular medium.

In conclusion, this study provides new insights about the possible interacting network of the various proteins involved in iron release from PVDI in the periplasm of *P. aeruginosa* (Fig. 8). These interactions have been highlighted using a two-hybrid approach and confirmed *in vitro* using purified or pulldown experiments; but they existence still need to be confirmed in *P. aeruginosa* cells. This complex interacting network strongly suggests a multi-protein complex at the inner membrane, allowing iron to be removed from PVDI. Consequently, the molecular mechanisms for iron acquisition via PVDI involves the following steps (Fig. 8D). As detailed in the introduction, PVDI-Fe^3+^ is imported across the outer membrane into the bacterial periplasm by FpvAI and FpvB. In the periplasm, PVDI forms a FpvC-FpvF-PVDI-Fe complex with the two periplasmic-binding proteins. As FpvJ was found to interact with both periplasmic and membrane proteins, it may help in the interaction of this periplasmic complex with the inner-membrane FpvG-FpvH complex. Iron reduction by FpvG decreases the affinity of PVDI for the metal and a transfer of iron to the periplasmic-binding protein FpvC, which likely brings iron to the FpvDE ABC transporter. Apo-PVDI is most likely bound to FpvF, which is able to interact with PvdT, allowing the recycling of apo-PVDI to the extracellular medium by the efflux pump PvdRTOpmQ. Moreover, this FpvGHJCF complex is the first example to be described of a complex between an inner-membrane reductase and two periplasmic-binding proteins associated with an ABC transporter.

Deciphering and understanding the protein-protein interacting network is an important piece in the understanding of the PVDI-Fe^3+^ uptake pathway puzzle. Our work will undoubtedly initiate a number of future directions like chemical crosslinking experiments in *P. aeruginosa* cells with tagged proteins to assess the existence of this interacting network in the pathogen. Electron microscopy approaches are planned to obtain structural information on these different protein complexes. At last, further studies are also needed to understand the exact role of FpvH, FpvJ, and FpvK within the complexes and PVDI-Fe dissociation.

## Materials and Methods

### Chemicals, bacterial strains and growth medium

Medium culture Lysogeny Broth (LB) and LB-agar were purchased from Difco. Detergent n-dodecyl-ß-D-maltoside (DDM) was purchased from Anatrace, N-Lauroylsarcosine sodium (SLS) and Tween 20 from Sigma. The strains used in this study are listed in the Supplementary Table S1. Briefly, TOP10 and DH5α strains were used for cloning procedures, TOP10 and BL21 strains for protein production and DHM1 strains for bacterial two-hybrid assays. *E. coli* strains were routinely grown in LB medium at 37 °C and on LB-agar for solid culture. Plasmids were maintained by the addition of antibiotics such as ampicillin (100 µg/ml), kanamycin (50 µg/ml), chloramphenicol (50 µg/ml) and streptomycin (100 µg/ml).

### Plasmid construction

Plasmids used in this study are listed in Supplementary Table S1. All the PCRs were performed with DNA Phusion high-fidelity polymerase (Thermofischer Scientific). DNA sequences from *Pseudomonas aeruginosa* PAO1 were taken from Pseudomonas Genome DataBank (www.pseudomonas.com). Oligonucleotides were purchased from Sigma and are listed in Supplementary Table S2. All primers used introduced restriction sites.

#### Bacterial two-hybrid vectors

pKT25 and pUT18C vectors were used for the expression of membrane proteins, whereas pKTM25-zip and pUTM18C vectors were used for the periplasmic proteins. Coding regions of *fpvG, fpvH, fpvK, fpvE*, *fpvD and pvdT* were amplified by PCR with primer pairs 1159/1160, 1161/1162, 1177/1178, 1199/1200, 1197/1198 and 1420/1411 respectively. The PCR products were digested with *KpnI*/*XbaI* and inserted into the same sites of pKT25 or pUT18C vectors to create pKT25-FpvG, pKT25-FpvH, pKT25-FpvK, pKT25-FpvD, pKT25-FpvE, pUT18C-FpvG, pUT18C-FpvH, pUT18C-FpvK, pUT18C-FpvD, pUT18C-FpvE and pKT25-PvdT. Coding regions of *fpvJ* and *fpvC* were amplified with primer pairs 1397/1201 and 1408/1202 respectively, and the relevant PCR products were digested with *XbaI*/*KpnI* or *XbaI*/*SacI* and cloned into the same sites of pKTM25-zip to create pKTM25-FpvJ and pKTM25-FpvC. To clone into the pUTM18C vector, *fpvJ*, *fpvC* and *fpvF* genes were amplified with primer pairs 1397/1212, 1408/1400 and 1399/1203 respectively, and then digested with *XbaI*/*KpnI* to be inserted into the same restriction sites of pUTM18C to create pUTM18C-FpvJ, pUTM18C-FpvC and pUTM18C-FpvF.

#### Protein production vectors

To construct pBAD24-FpvG_His6_ and pBAD33-FpvH_Strep_ vectors, coding regions of *fpvG* and *fpvH* were amplified with primer pairs 1185/1186 and 1189/1190, respectively. PCR products of *fpvG* and *fpvH* were digested with *NcoI*/*HindIII* and *EcoRI*/*HindIII* and inserted into the same sites of pBAD24 or pBAD33. To clone into the pRSF and pCDF vectors, primer pairs 1404/1405, 1408/1409 and 1406/1407 were used to amplify *fpvJ*, *fpvC* and *fpvF*, respectively. PCR products were digested by *EcoRI*/*HindIII* for *fpvJ* and *fpvC*, and *BglII*/*KpnI* for *fpvF* and were cloned into the same restriction sites of pRSF or pCDF vectors to create pCDF-FpvJ_His6_, pCDF-FpvJ_His6_FpvF_Flag_, pRSF-FpvC_HA_ and pCDF-FpvF_Flag_.

All constructs were screened with colony PCR and plasmids were purified with the Macherey Nagel Nucleospin Plasmid kit in accordance with the manufacturer’s instructions. All constructions were verified by DNA sequencing (Eurofins).

### Bacterial two-hybrid assay

For plate-BACTH assay, two compatible vectors producing proteins fused to T18 or T25 domain were co-transformed into DHM1 cells that were incubated at 30 °C for 16 h. Ten independent colonies of each transformation were inoculated together into 2 ml of LB medium supplemented with ampicillin, kanamycin and 0.5 mM isopropyl β-D-1-thiogalactopyranoside (IPTG, Sigma) and incubated at 30 °C for 16 h. 5 µl of each culture was spotted onto LB-agar plate supplemented with appropriate antibiotics, 0.5 mM IPTG and 40 µg/ml 5-bromo-4-chloro-3-indolyl-β-D-galactopyranoside (X-gal, Sigma). The plate was incubated for 16 h at 30 °C.

For liquid medium assay, two compatible vectors producing proteins fused to T18 or T25 domain were co-transformed into DHM1 cells that were incubated at 37 °C for 16 h. Ten independent colonies of each transformation were inoculated into 2 ml of LB-medium supplemented with appropriate antibiotics and were incubated at 37 °C during 24 h. The next day, 20 µl of each culture were inoculated in 2 ml of LB supplemented with appropriate antibiotics and 0.5 mM IPTG and incubated at 37 °C for 16 h. 100 µl of each culture was used for the ß-galactosidase assay using Miller Protocol^36^.

### ß-galactosidase dosage

100 µl of bacterial culture were added to 900 µl of Z Buffer (60 mM Na_2_HPO_4_, 40 mM NaH_2_PO_4_,10 mM KCl, 1 mM MgSO_4_, pH 7.0, 0.2% β-mercaptoethanol). 1 µl of 0.1% sodium dodecylsulfate and 50 µl of chloroform were added to the suspension that was mixed vigorously for 10 seconds. The suspension was then incubated for 5 min at 28 °C. 200 µl of 4 mg/ml 2-nitrophenyl ß-D-galactopyranoside (ONPG, Sigma) were added to the cells. Reaction was stopped by adding 500 µl of 1 M Na_2_CO_3_. The suspension was centrifuged at 14,000 g for 3 min and the optical density of the supernatant was read at 420 and 550 nm. The ß-galactosidase activity was then calculated in Miller Unit (MU) according to the following equation:

$${\rm{MU}}=\frac{1000\times ({{\rm{OD}}}_{420{\rm{nm}}}-1.75\,\times {{\rm{OD}}}_{550{\rm{nm}}})}{{\rm{Time}}\,({\rm{\min }})\times {\rm{Volume}}({\rm{mL}})\times {{\rm{OD}}}_{600{\rm{nm}}}}$$

### Expression and purification of the FpvG-FpvH inner membrane complex

*E. coli* TOP10 strain was co-transformed with pBAD24-FpvG_His6_ and pBAD33-FpvH_Strep_. Overnight culture was inoculated into LB medium supplemented with ampicillin and chloramphenicol and grown at 37 °C until OD_600nm_ reached 0.6. Then, protein production was induced by addition of 0.01% L-arabinose for 4 h at 30 °C. Cell pellet was re-suspended in 50 mM Tris-HCl pH 8.0, 100 mM NaCl and one protease inhibitor tablet (complete EDTA-Free protease inhibitor, Roche). After sonication, unbroken cells were removed by centrifugation at 12,000 g for 15 min at 4 °C. The supernatant was ultracentrifuged during 40 min at 100,000 g at 4 °C. The pellet was re-suspended into 50 mM Tris-HCl pH 8.0, 50 mM NaCl, 1% DDM and solubilized overnight at 4 °C. Membranes were collected by ultracentrifugation at 100,000 g for 40 min at 4 °C and loaded onto a StrepTrap Column (GE Healthcare) equilibrated with Buffer A (50 mM Tris-HCl pH 8.0, 50 mM NaCl, 0.1% DDM). The FpvG-FpvH complex was eluted with Buffer A supplemented with 2.5 mM D-Desthiobiotin (Sigma). Fractions of interest were then concentrated using a 10,000 kDa molecular weight cut off (Amicon, Millipore) and loaded onto a Superdex 200 10/300 GL column equilibrated with 50 mM Tris-HCl pH 8.0, 50 mM NaCl, 0.025% DDM. Fractions containing FpvH_Strep_ and FpvG_His6_ were collected for further analysis.

### Expression of the FpvJ-FpvF-FpvC periplasmic complex

BL21(DE3) cells were transformed with pCDF-FpvJ_His6_-FpvF_Flag_ and pRSF-FpvC_HA_. Overnight culture was inoculated into LB medium with streptomycin and kanamycin and grown at 37 °C until OD_600_ reached 0.6. Then, protein production was induced by adding 0.4 mM IPTG at 22 °C for 16 h. Cells were subjected to cellular fractionation as described below for further analysis.

### Immunoblot analysis

Nitrocellulose membranes were used for protein transfer by electroblotting. After saturation with blocking buffer (5% dried-milk powder, PBS 1X, 0.1% Tween 20), the membranes were incubated overnight at 4 °C with primary antibody: anti-Strep (1/2000, IBA), anti-HA (1/2000, Sigma), anti-FpvC (laboratory collection), anti-His_6_ (1/3000, GeneTex), anti-OmpC (Biorbyt), anti-LepB (Biorbyt), anti-Ef-Tu (LSBio) or anti-MBP (Bioss antibodies). Immunoblots were developed by using horseradish peroxidase-conjugated goat anti-rabbit or anti-mouse antibodies (1/10000, GE Healthcare) followed by chemiluminescence detection. Molecular mass marker was purchased from Thermofischer Scientific.

### Cellular fractionation

#### Periplasm and cytoplasm isolation

*E. coli* strains overproducing proteins of interest were pelleted, washed with 50 mM Tris-HCl pH 8.0 and re-suspended into Tris-Sucrose EDTA Buffer (0.2 M Tris-HCl pH 8.0, 20% sucrose and 1 mM EDTA). 200 µg/ml of lysozyme were added to the suspension, incubated for 1 h at 4 °C and the cells were centrifuged at 6,700 g for 10 min at 4 °C to remove unbroken cells and insoluble fraction (like insoluble proteins). The supernatant corresponding to the periplasm was ultracentrifuged at 100,000 g for 40 min at 4 °C. Spheroplasts were washed three times with Tris-Sucrose buffer and re-suspended into chill water and treated with benzonase (Sigma, 250U/µl). After incubation for 1 h at 37 °C, suspension was centrifuged for 40 min at 100,000 g at 4 °C to collect the cytoplasm (supernatant). The pellet, corresponding to the total membranes, was re-suspended in 50 mM Tris-HCl pH 8.0.

#### Membrane isolation

*E. coli* strains overproducing proteins of interest were pelleted, re-suspended in 50 mM Tris-HCl pH 8.0 and lysed by sonication. Unbroken cells were removed by centrifugation at 12,000 g for 15 min. Supernatant was centrifuged at 100,000 g for 40 min. The membranes (pellet) were solubilized in 50 mM Tris pH 8.0, 100 mM NaCl, 0.1% SLS for 16 h at 4 °C and ultracentrifuged at 100,000 g for 40 min at 4 °C. The pellet corresponds to the outer membranes and the supernatant to the inner membranes.

### Pulldown experiments

#### Periplasmic proteins

BL21 (DE3) cells were transformed with pCDF-FpvJ_His6_-FpvF_Flag_ and pRSF-FpvC_HA_ or with pCDF-FpvF_Flag_ or pRSF-FpvC_HA_ only. Overnight culture was inoculated into LB medium with appropriate antibiotics and grown at 37 °C until OD_600_ reached 0.6. Then protein production was induced by adding 0.4 mM IPTG at 22 °C for 16 h. Cells were re-suspended in buffer A (50 mM Tris pH 8.0, 250 µM EDTA, 20% sucrose), subjected to cellular fractionation and the periplasmic fraction was recovered. 100 µl of the periplasmic fraction were mixed with 50 µl agarose beads charged with nickel (Sigma) and incubated on a rotating wheel for 1 h at room temperature. The mixture was then centrifuged 2 min at 2,000 rpm to remove the unbound proteins. Beads were washed twice with 50 mM Hepes pH 7.5, 50 mM NaCl before being recovered by centrifugation for further analysis.

#### Periplasmic and membrane proteins

Periplasmic fractions containing FpvJ_His6_, FpvF_Flag,_ FpvC_HA_ were incubated with solubilized membranes isolated from TOP10 cells producing FpvG_His6_ and FpvH_Strep_ proteins and submitted to anti-Flag resin (Sigma). The next steps were performed as described above. When the experiment has been carried out in the presence of PVDI-Fe, 10 µM of the ferri-siderophore complex have been added during incubation with the anti-Flag resin.

## Supplementary information

SupplementaryInformation
