# Supplementary material for: Iron acquisition in Pseudomonas aeruginosa by the siderophore pyoverdine: an intricate interacting network including periplasmic and membrane proteins
Source: Sci Rep. 2020 Jan 10;10:120. doi: 10.1038/s41598-019-56913-x (PMC6954188; doi:10.1038/s41598-019-56913-x)
Supplement: Supplementary file 1 — SupplementaryInformation [file 41598_2019_56913_MOESM1_ESM.pdf]

SUPPLEMENTARY MATERIAL

Iron acquisition in *Pseudomonas aeruginosa* by the siderophore pyoverdine: an intricate interacting network including periplasmic and membrane proteins

Anne Bonneau[a,b], Béatrice Roche[a,b*], and Isabelle Schalk[a,b*]

Strains		Caracteristics	Reference
E. coli TOP10		F-, *mcrA* Δ(*mrr-hsdRMS-mcrBC*), ϕ80*lacZ*, Δ*M15*, Δ*lacX74*, *recA1*, *araD139*, Δ(*ara-leu*)7697, *galU*, *galK*, *rpsL*, *endA1*, *mup*	Invitrogen
E. coli BL21 (DE3)		F^-, *ompT*, *gal*, *dcm*, *lon*, $hsdS_B(r_B^- m_B^-)$, λ(DE3 [*lacI*, *lacUV5-T7p07, ind1, sam7, nin5*]), $[malB^+]_{K-12}(\lambda^S)$	Novagen
E. coli DH5α		F- Δ(*lacZYA-argF*)U169, *recA1*, *endA1*, *hsdR17*, *thi-1*, *gyrA96*, r*elA*1	Laboratory collection
E. coli DHM1		F-, cya-, *recA1*, *endA*, *gyrA96, thi-1, hsdR17, spoT1, rfbD1, cya-854*	[1]
**Plasmids**	**ID collection**	**Caracteristics**	**Reference**
pKT25	283	P15 origin, Plac, N-terminal T25 cyclase fragment, Kan^R	[1]
pUT18C	282	ColE1 origin, Plac, N-terminal T18 cyclase fragment, Ap^R	[1]
pUTM18C	304	Modified pUT18C with the first TM domain of OppB (*E. coli*) inserted downstream T18	[2]
pUTM18C-zip	305	Modified pUT18C with the first TM domain of OppB (*E. coli*) inserted downstream T18, encoding a fragment of leucine zipper protein	[2]
pKTM25-zip	306	Modified pKT25 with the first TM domain OppB (*E. coli*) inserted downstream T25, encoding a fragment of leucine zipper protein	[2]
pKT25-FpvG	pAB9	FpvG cloned into pKT25, downstream T25 sequence	This study
pKT25-FpvH	pAB10	FpvH cloned into pKT25, downstream T25 sequence	This study
pKT25-FpvK	pAB11	FpvK cloned into pKT25, downstream T25 sequence	This study
pKT25-pvdT	pAB24	pvdT cloned into pKT25, downstream T25 sequence	This study
pKT25-FpvD	pAB25	FpvD cloned into pKT25, downstream T25 sequence	This study
pKT25-FpvE	pAB26	FpvE cloned into pKT25, downstream T25 sequence	This study
pKT25-RetS	285	RetS cloned into pKT25, downstream T25 sequence	[3]
pUT18C-FpvG	pAB12	FpvG cloned into pUT18C, downstream T18 sequence	This study
pUT18C-FpvH	pAB13	FpvH cloned into pUT18C, downstream T18 sequence	This study
pUT18C-FpvK	pAB14	FpvK cloned into pUT18C, downstream T18 sequence	This study
pUT18C-FpvD	pAB27	FpvD cloned into pUT18C, downstream T18 sequence	This study
pUT18C-FpvE	pAB28	FpvE cloned into pUT18C, downstream T18 sequence	This study
pUT18C-RetS	284	RetS cloned into pUT18C, downstream T18 sequence	[3]
pKTM25-FpvJ	pAB19	FpvJ cloned into pKTM25-zip vector	This study
pKTM25-FpvC	pAB20	FpvC cloned into pKTM25-zip vector	This study
pUTM18C-FpvC	pAB22	FpvC cloned into pUTM18C	This study
pUTM18C-FpvF	pAB23	FpvF cloned into pUTM18C	This study
pUTM18C-FpvJ	pAB21	FpvJ cloned into pUTM18C	This study
pBAD24	348	pBR322 origin, Para, araC, Ap^R	[4]
pBAD33	349	pACYC184 origin, Para, araC, Cm^R	[4]
pRSFDuet-1	253	RSF origin, pT7, Kan^R	Novagen
pCDFDuet-1	357	CDF origin, pITG, lacI, $Strep^R$	Novagen

pBAD24-FpvG_{His6}	pAB2	FpvG cloned into pBAD24 with C-terminal His_6 sequence	This study
pBAD33-FpvH_{Strep}	pAB3	FpvH cloned into pBAD33 with C-terminal Strep sequence	This study
pCDF-FpvJ_{His6}FpvF_{Flag}	pAB6	FpvJ and FpvF cloned into MCS1 of pCDFDuet-1with C-terminal His_6 and Flag sequence, respectively.	This study
pCDF-FpvJ_{His6}	pAB5	FpvJ cloned into MCS1 of pCDFDuet-1 with C-terminal His_6 sequence	This study
pRSF-FpvC_{HA}	pAB7	FpvC cloned into MCS2 of pRSFDuet-1 with C-terminal HA sequence	This study
pCDF-FpvF_{Flag}	pAB8	FpvF cloned into MCS1 of pCDFDuet-1 with C-terminal Flag sequence	This study

Table S1 Strains and plasmids used in this study

**Two-hybrid plasmids**	**ID collection**	**Sequence (5' → 3')**
pKT25-FpvGUP	1159	CCGGTCTAGAGATGTCGAAAAAGTCCCGCTCCAGA
pKT25-FpvGDO	1160	CCGGGGTACCCGTGGCTGTCCCTCCGCGGTCTGGTT
pKT25-FpvHUP	1161	CCGGTCTAGAGATGAGCAAGGCCGCCGTTGCCCAG
pKT25-FpvHDO	1162	CCGGGGTACCCGTTGCTTACCTCGTTTGTTCAGCCA
pKT25-FpvKUP	1177	CCGGTCTAGAGATGAGCGTCGAAACCGTCCGCCCG
pKT25-FpvKDO	1178	CCGGGTACCCGATGGCGGTGCTGACGGGTCACGCC
pKT25-FpvDUP	1197	CCGGTCTAGAGATGAGCATCCACACCCGCCCCTGG
pKT25-FpvDDO	1198	CCGGGGTACCCGTGGCGCGGCGATGTCGGCGTGGTC
pKT25-FpvEUP	1199	CCGGTCTAGAGATGAACGACGCCTACGAACAACTG
pKT25-FpvEDO	1200	CCGGTCTAGAGATGAACGACGCCTACGAACAACTG
pKT25-pvdTUP	1410	CCGGTCTAGAGATGGAAAACGCCACGCAACCCGTC
pKT25-pvdTDO	1411	CCGGGGTACCCGTTGGCTGGCCAGGGCGGCCACCGG
pKTM25-FpvCUP	1408	CCGGTCTAGAGGAAGACGGCAAACGCCTGCGCATC
pKTM25-FpvCDO	1202	CCGGGAGCTCTCATGCGCCGGACTCCTGGATGGC
pUTM18C-FpvCDO	1400	CCGGGGTACCCGTGCGCCGGACTCCTGGATGGCGCG
pKTM25-FpvJUP	1397	CCGGTCTAGAGCACAACCCGATCTGCGAATGCGAG
pKTM25-FpvJDO	1201	CCGGGAATTCTCATGGCGCGGCGATGTCGGCGTG
pUTM18C-FpvJDO	1212	CCGGGGTACCCGTGGCGCGGCGATGTCGGCGTGGTC
pUTM18C-FpvFUP	1399	CCGGTCTAGAGGCGACGCCGGCCGCGGCCCAGTCC
pUTM18C-FpvFDO	1203	CCGGGGTACCCGTCAGCCCTTACCGGCCAGCAGCCC
**Proteins production plasmids**		**Sequence (5' → 3')**
FpvG-p24HisCter_F	1185	CCGGCCATGGCCTCGAAAAAGTCCCGCTCCAGACTC
FpvG-p24HisCTer_R	1186	CCGGAAGCTTTCAATGATGATGATGATGATGGCTAGCTGGCTGTCCCTCCGCGGTCTGGTT
FpvH-p33StrepCter_F	1189	CCGGGAATTCAAGGAGATATACATATGAGCAAGGCCGCCGTTGCCCAGCCC
FpvH-p33StrepCter_R	1190	CCGGAAGCTTTCATTTTTCGAACTGCGGGTGGCTCCAGCTAGCTTGCTTACCTCGTTTGTT CAGCCA
FpvJ-pcDFHisCter_F	1404	CCGGCCATGGGCAGCATCCACACCCGCCCCTGGCGC
FpvJ-pCDFHisNter_R	1405	CCGGGAATTCTCAATGATGATGATGATGATGGCTAGCTGGCGCGGCGATGTCGGCGTGGT C
FpvF-pCDFFlagCter_F	1406	CCGGGAATTCGAAGGAGATATACATATGAACCTGAAACCCCATTGCCTC
FpvF-pCDFFlagCter_R	1407	CCGGAAGCTTTCACTTGTCGTCATCGTCTTTGTAGTCGCTAGCGCGCCCTTCGACTATCGC TTTCTGT
FpvC-pRSFHACter_F	1408	CCGGAGATCTAAGGAGATATACATATGCTGTTCTCCCGTCGCTCATCC
FpvC-pRSFHACter_R	1409	CCGGGGTACCTCAAGCGTAATCTGGAACATCGTATGGGTAGCTAGCTGCGCCGGACTCCT GGATGGCGCG

Table S2: Oligonucleotides used in this study

Protein	Theoretical MW (KDa)	Theoretical MW with tag (KDa)
**FpvG**	45	46
**FpvH**	20	21
**FpvJ**	11	15
**FpvF**	32	33
**FpvC**	34	36

Table S3: Theoretical MW of the proteins tested for protein interactions in this study with and without tag.

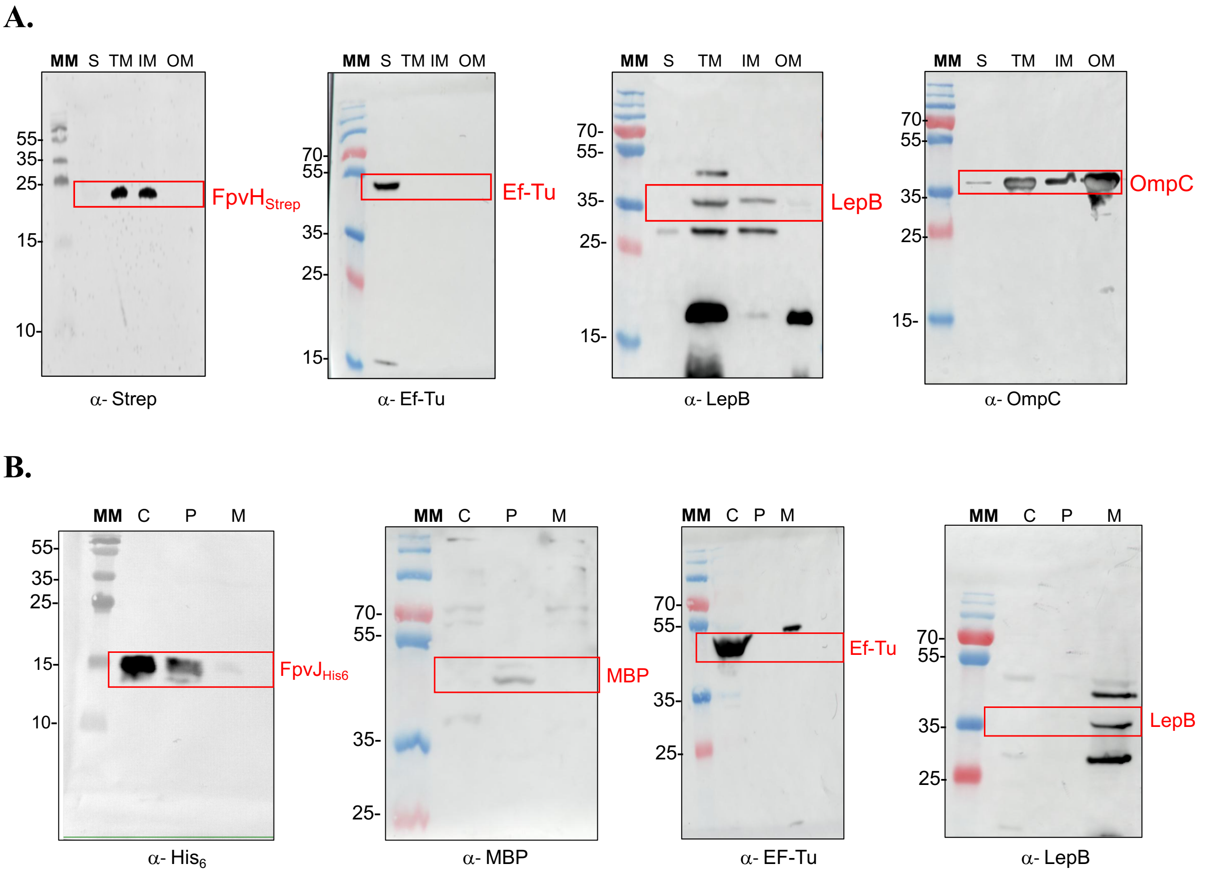

Fig. S1. (A) TOP10 cells producing $FpvH_{Strep}$ were harvested and membrane fractions were collected as described in Materials and Methods procedures. Samples were loaded onto a 12 % SDS-PAGE gel for immunodetection analysis. MM, molecular marker; S, soluble fraction; TM, Total membrane; IM, Inner membranes; OM, Outer membranes. To check for correct cell fraction antibody against specific cytoplasmic, inner and outer membrane proteins were used: anti-Ef-Tu for the soluble fraction (Ef-Tu

being a cytoplasmic protein); anti-LepB for the inner membrane fraction and anti-OmpC for the outer membrane fraction. Anti-Strep was used to detect $FpvH_{Strep}$.
(B) BL21 (DE3) cells producing $FpvJ_{His6}$ were submitted to cellular fractionation. MM, molecular marker; P, periplasm, C, cytoplasm, TM, total membranes were collected and loaded onto a 15 % SDS-PAGE gel for immunodetection analysis. The molecular mass markers (kDa) are indicated on the left. To check for correct cell fraction antibody against specific cytoplasmic, periplasmic and membrane proteins were used: anti-Ef-Tu for the cytoplasmic fraction; anti-MBP for the periplasmic fraction and anti-LepB for the membrane fraction. Anti-His was used to detect $FpvJ_{His}$.
Ef-Tu is a cytoplasmic elongation factor, MBP (maltose binding protein) is a periplasmic binding protein involved in the uptake of maltodextrins, LepB an inner membrane peptidase, OmpC an outer membrane porin.

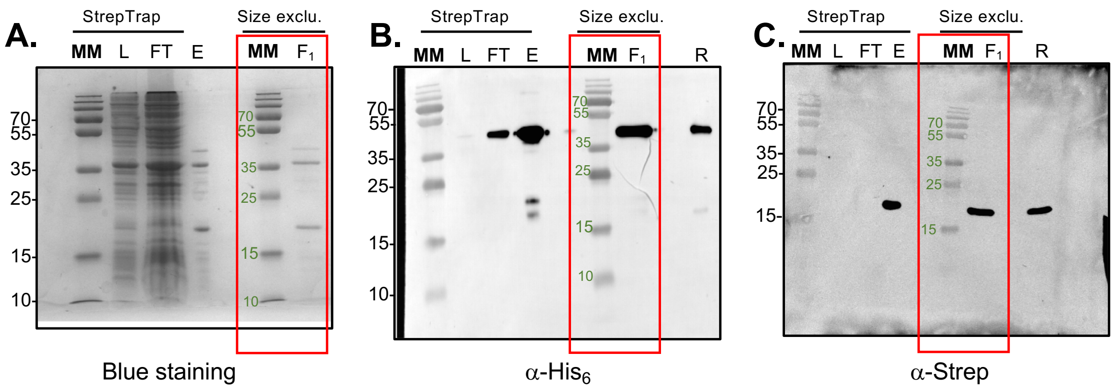

Fig. S2: Original gels of SDS-PAGE and blots presented in Fig. 3. FpvG-FpvH was first purified on a StrepTrap column and afterwards on a Size exclusion chromatography (Superdex 200 10/300 GL column). (A) SDS-PAGE of the purified complex analyzed by Coomassie blue staining. (B-C) Immunoblot analysis using specific anti-His (B) and anti-Strep antibodies (C). The molecular mass markers (kDa) are indicated on the left. MM, Molecular Marker (KDa); L, Load fraction loaded on the affinity Strep column; FT, Flow-through; E: Elution (containing FpvG and FpvH); GF: Fraction of the size-exclusion chromatography containing FpvG and FpvH; R, Positive control with bacteria expressing His-tagged FpvG (panel B) and Strep tag FpvH (panel C). In red are the fractions of SDS-PAGE and blots presented in Fig. 3. The predicted size of $FpvG_{His6}$ and $FpvH_{Strep}$ are 46 kDa and 21 kDa respectively.

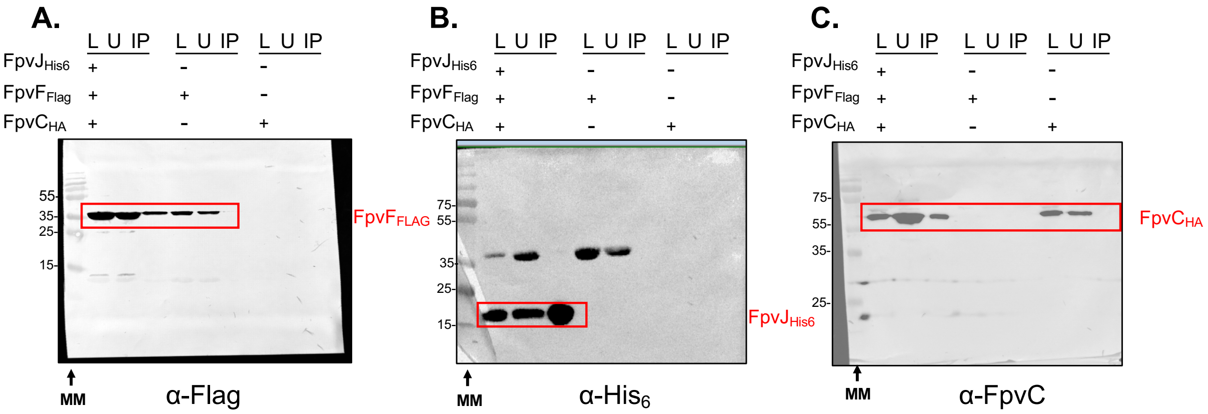

Fig. S3: Original gels of blots presented in Figure 5. Periplasmic fractions of BL21(DE3) cells co-expressing the indicated proteins $FpvJ_{His6}$, $FpvC_{HA}$, or $FpvF_{Flag}$ were incubated with anti-His beads. The loading (L), unbound (U), and immunoprecipitated (IP) fractions were separated by SDS-PAGE and analyzed by immunodetection using anti-Flag, anti-His_6 and anti-FpvC antibodies. The molecular mass markers (MM) are indicated on the left (kDa). In red are the fractions of blots presented in Figure 5. The predicted size of $FpvJ_{His6}$, $FpvF_{Flag}$ and $FpvC_{HA}$ are 15 kDa, 33 kDa and 36 kDa respectively. $FpvJ_{His6}$ expressed in BL21(DE3) cells, copurifies with a unknown protein of around 37 kDa.

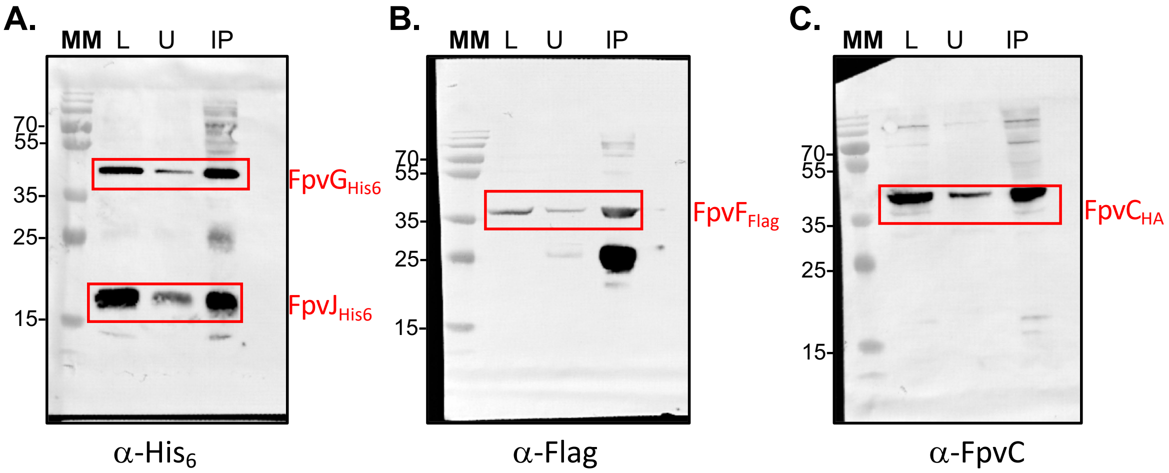

Fig. S4: Original gels of blots presented in Figure 6. Periplasmic fraction of BL21(DE3) cells and membrane fractions of TOP10 cells producing the indicated proteins $FpvJ_{His6}$, $FpvC_{HA}$, $FpvF_{Flag}$, $FpvG_{His6}$, and $FpvH_{Strep}$ were incubated with anti-Flag beads. The loading (L), unbound (U), and immunoprecipitated (IP) fractions were separated by SDS-PAGE and analyzed by immunodetection using anti-His_6 (panel A), anti-Flag (panel B) and anti-FpvC (panel C) antibodies. The molecular mass markers (MM) are indicated on the left (kDa). The predicted size are for $FpvG_{His6}$ 46 kDa, $FpvH_{Strep}$ 21 kDa, $FpvJ_{His6}$ 15 kDa, $FpvF_{Flag}$ 33 kDa and $FpvC_{HA}$ 36 kDa. In red are the fractions of blots presented in Figure 6.

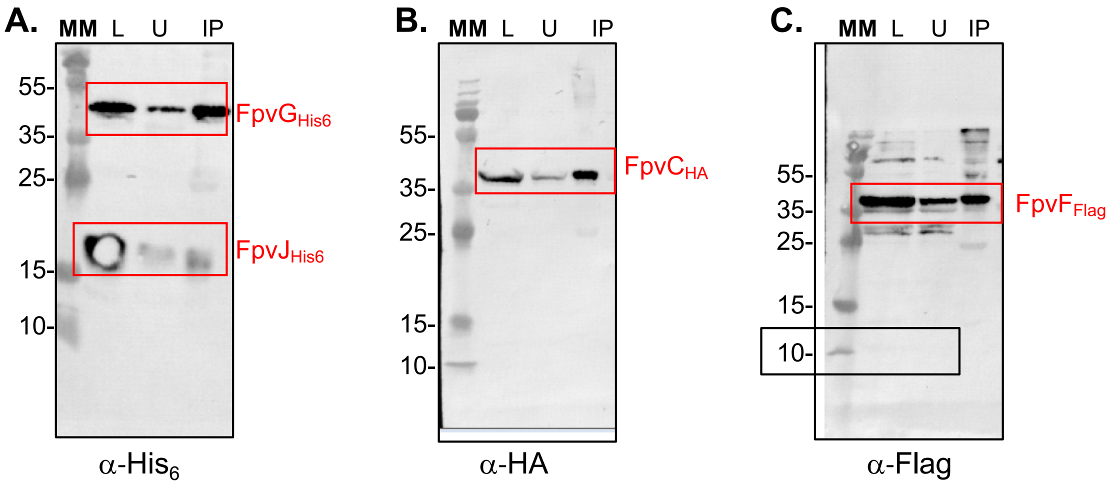

Fig. S5. Pulldown assay of the FpvG-FpvJ-FpvC-FpvF complex in the presence of 10 µM PVDI-Fe. Periplasmic fractions of BL21(DE3) cells and membranes fractions of TOP10 cells producing the indicated proteins FpvJ$_{His6}$, FpvC$_{HA}$, FpvF$_{Flag}$, FpvG$_{His6}$, and FpvH$_{Strep}$ were incubated with anti-Flag beads as in Figure 6 except that 10 µM PVDI-Fe was added. The loading (L), unbound (U), and immunoprecipitated (IP) fractions were separated by SDS-PAGE and analyzed by immunodetection using anti-His$_6$, anti-FpvC, and anti-Flag antibodies. The molecular mass markers (kDa) are indicated on the left. The predicted size are for FpvG$_{His6}$ 46 kDa, FpvH$_{Strep}$ 21 kDa, FpvJ$_{His6}$ 15 kDa, FpvF$_{Flag}$ 33 kDa and FpvC$_{HA}$ 36 kDa.

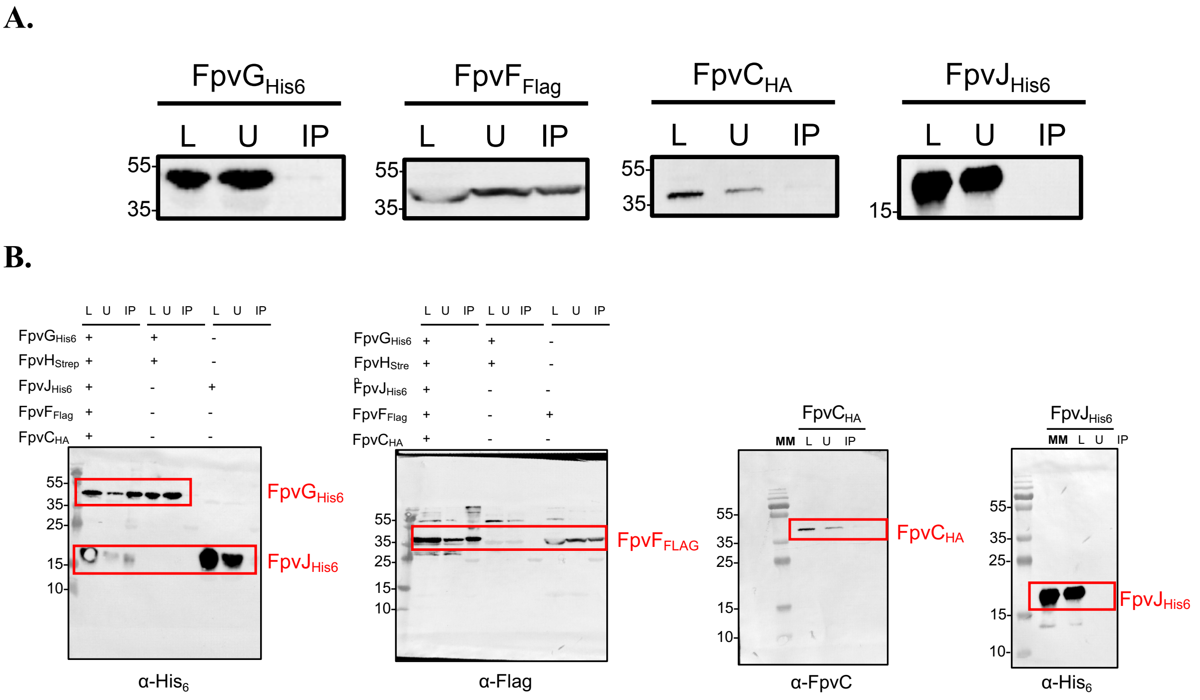

Fig. S6: (A) Negatives controls of the pull down assay presented in Fig. 6: periplasmic fractions of BL21(DE3) cells and membranes fractions of TOP10 cells producing the indicated proteins FpvF$_{Flag}$, FpvC$_{HA}$, FpvJ$_{His6}$ or FpvG$_{His6}$ were incubated with anti-Flag resin. The load (L), unbound (U) and immunoprecipitated (IP) fractions were separated by SDS-PAGE and analyzed in immunodetection by using anti-His$_6$, anti-FpvC and anti-Flag antibodies. (B)

Originals gels presented in panel A and in the Fig S5. For both panels, the molecular mass markers (MM) are indicated on the left in kDa. The predicted size are for $FpvG_{His6}$ 46 kDa, $FpvJ_{His6}$ 15 kDa, $FpvF_{Flag}$ 33 kDa and $FpvC_{HA}$ 36 kDa.

References

1. Karimova, G., Dautin, N. & Ladant, D. Interaction network among *Escherichia coli* membrane proteins involved in cell division as revealed by bacterial two-hybrid analysis. *J. Bacteriol.* **187**, 2233–2243 (2005).
2. Ouellette, S. P., Gauliard, E., Antosová, Z. & Ladant, D. A Gateway(®) -compatible bacterial adenylate cyclase-based two-hybrid system. *Environ Microbiol Rep* **6**, 259–267 (2014).
3. Vincent, F. *et al.* Distinct oligomeric forms of the *Pseudomonas aeruginosa* RetS sensor domain modulate accessibility to the ligand binding site. *Environ. Microbiol.* **12**, 1775–1786 (2010).
4. Guzman, L. M., Belin, D., Carson, M. J. & Beckwith, J. Tight regulation, modulation, and high-level expression by vectors containing the arabinose PBAD promoter. *J Bacteriol* **177**, 4121–4130 (1995).
